# Implementing systematic melanoma risk assessment and risk‐tailored surveillance in a skin cancer focussed dermatology clinic: A qualitative study of feasibility and acceptability to patients and clinic staff

**DOI:** 10.1002/cam4.6976

**Published:** 2024-02-04

**Authors:** A. L. Smith, A. K. Smit, B. I. Laginha, N. Singh, B. Gallo, L. Martin, A. E. Cust

**Affiliations:** ^1^ The Daffodil Centre The University of Sydney, a joint venture with Cancer Council NSW Sydney New South Wales Australia; ^2^ Melanoma Institute Australia, The University of Sydney Sydney New South Wales Australia; ^3^ Faculty of Medicine and Health, Sydney School of Public Health The University of Sydney Sydney New South Wales Australia; ^4^ Australian Institute of Health Innovation, Macquarie University Sydney New South Wales Australia; ^5^ Faculty of Medicine and Health University of New South Wales Sydney New South Wales Australia

**Keywords:** early detection, implementation science, melanoma, risk assessment, screening, skin cancer, surveillance

## Abstract

**Background:**

International bodies recommend that melanoma risk assessment should be integrated into skin cancer care provision, but evidence to support implementation is lacking.

**Aim:**

To explore the acceptability and feasibility of implementing personalised melanoma risk assessment and tailored patient education and skin surveillance within routine clinical care.

**Methods:**

This prospective qualitative implementation study was informed by the Theoretical Framework of Acceptability (TFA). Personalised, systematic melanoma risk assessment was implemented in the dermatology clinic at the Melanoma Institute Australia, Sydney, Australia February–May 2021. Pre‐ and post‐implementation observations and semi‐structured interviews with patients and staff were conducted (September 2020–March 2021). Observational notes and interview transcript data were analysed thematically using the TFA as a classifying framework.

**Results:**

A total of 37 h of observations were made, and 29 patients and 12 clinic staff were interviewed. We found that the delivery of personalised melanoma risk estimates did not impact on patient flow through the clinic. Dermatologists reported that the personalised risk information enhanced their confidence in assessing patient risk and recommending tailored surveillance schedules. Most patients reported that the risk assessment and tailored information were a beneficial addition to their care. Among patients whose risk deviated from their expectations, some reported feeling worried, confused or mistrust in the risk information, including those at lower risk who were recommended to decrease surveillance frequency.

**Conclusions:**

It is feasible and acceptable to patients and clinic staff to calculate and deliver personalised melanoma risk information and tailored surveillance as part of routine clinical care within dermatology clinics.

## 1 INTRODUCTION

Melanoma is an important challenge to cancer control globally.^1^ Early detection can minimise the impacts of melanoma as the stage of disease at diagnosis is associated with morbidity, mortality and treatment costs.^2^ Given the insufficient evidence that screening reduces melanoma mortality, guidelines emphasise targeting high‐risk groups for clinical skin examinations and behavioural counselling on preventive behaviours.^3^, ^4^

Tailoring melanoma prevention and early detection advice according to risk requires that healthcare professionals assess patients' risk of melanoma. However, there are no standardised risk assessment processes, and risk is often assessed intuitively based on individual risk factors.^5^ Validated melanoma risk prediction tools can enable clinicians to advise patients more accurately about their risk of developing a first or subsequent melanoma based on ultraviolet radiation exposure, phenotypic, genetic and histopathological risk factors.^6^, ^7^ Previous research has shown that a program of specialised surveillance for people at very high risk of melanoma (6‐monthly visits, total body photography and sequential digital dermoscopy imaging) resulted in earlier diagnosis and fewer excisions under a careful ‘wait‐and‐watch’ approach, and cost savings for the health system.^8^ Conversely, people at low risk of melanoma may benefit from fewer skin checks to reduce the harms of screening, including the potential for overdiagnosis.^9^

Systematic melanoma risk assessment using a validated risk prediction tool could enable the identification of risk groups in the patient population. It would also help ensure that high demand specialised clinical services such as those provided by dermatology clinics are used most efficiently. Incorporating systematic risk assessment and risk‐tailored skin surveillance into routine care will require changes to workflow and surveillance frequencies for some patients. Key considerations for the successful implementation, including feasibility and acceptability, of personal risk assessment and risk‐tailored surveillance are not currently well understood. Acceptability refers to the extent to which people delivering or receiving a healthcare intervention consider it to be appropriate, based on anticipated or actual cognitive and emotional responses to the intervention.^10^ Feasibility describes the actual fit or suitability of the intervention for everyday use.^11^

In a prospective implementation study conducted in a large dermatology clinic at the Melanoma Institute Australia in Sydney, Australia, we aimed to evaluate the acceptability and feasibility of implementing systematic personalised melanoma risk assessment and tailored patient education and skin surveillance and explore the associated barriers and facilitators. Our findings will inform implementation strategies for scaling up this approach and guide targeted skin cancer screening policy.

## 2 MATERIALS AND METHODS

### 2.1 Study design

Using mixed qualitative methods (semi‐structured interviews and observations), we aimed to explore the acceptability and feasibility of patients completing an iPad‐based validated melanoma risk assessment, receiving personal melanoma risk information and risk‐tailored skin surveillance recommendations alongside behavioural counselling on prevention and early detection behaviours within routine clinical care. This study was approved by Sydney Local Health District Ethics Review Committee (X18‐0426, HREC/18/RPAH/606), and all participants provided written informed consent.

### 2.2 Implementation strategy

**FIGURE 1:**
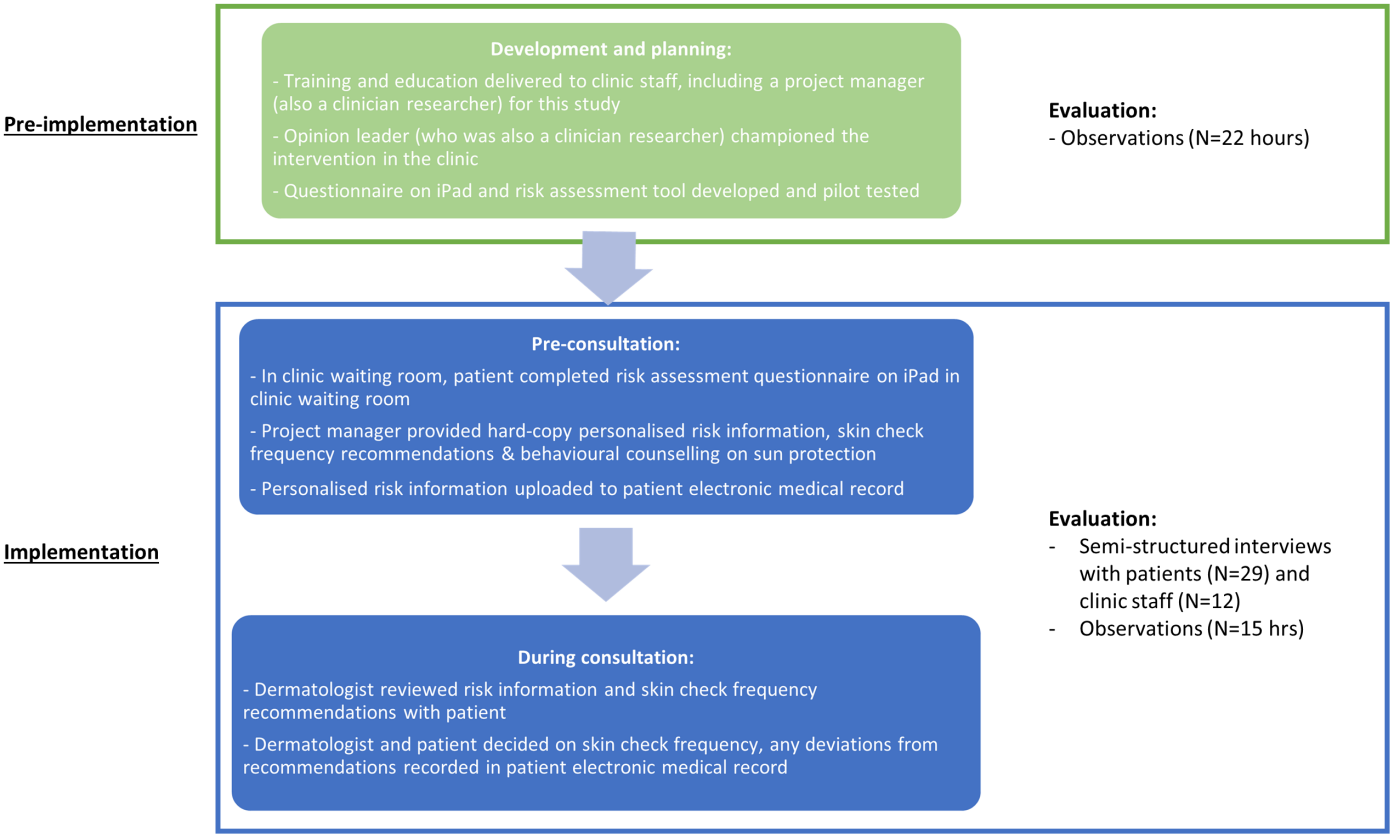
Study overview.

This study had two phases: (1) pre‐implementation (September 2020–January 2021) and (2) implementation (February–May 2021) (Figure 1). The observations in the dermatology clinic waiting room and consultation rooms and the patient interviews were conducted by ALS between September 2020 and March 2021. The staff interviews were conducted by ALS from November to December 2021.

#### 2.2.1 Phase 1: pre‐implementation

For the pre‐implementation phase, we drew on the implementation science literature to develop a strategy for successful integration. The project team included researchers (epidemiologist, statistician, implementation scientist, research assistant), clinicians (dermatologists and trainees), clinic support staff, data managers and software developers based at the Melanoma Institute Australia. The Institute is a tertiary referral centre and provides care for about one third of people who develop melanoma in New South Wales. People with and without a history of melanoma attend the dermatology clinic. In the study team, a project manager who was a clinician researcher conducted the majority of study processes in the clinic. The study team met fortnightly throughout the study, which provided a continuous feedback loop for addressing issues as they arose. During pre‐implementation meetings, processes and materials were reviewed and pilot tested by the study team.

The iPad‐based risk assessment survey incorporated questions from two online risk calculators that were based on validated published risk prediction models^6^, ^7^ (available at www.melanomarisk.org.au) and also included other relevant risk factor questions taken from other studies^12^ and clinical questions. For this study, a specific platform hosted by the Melanoma Institute Australia was established for the survey, which generated personal melanoma risk based on the published risk prediction models and securely stored all patient responses. The risk models use patients' risk factor information to estimate an individual's remaining lifetime risk of developing an initial primary invasive melanoma or, for those people who have previously been diagnosed with melanoma, their 10‐year risk of developing a subsequent primary invasive or in situ melanoma. For this study, we devised tailored recommendations whereby the frequency and healthcare setting (i.e., dermatology clinic or primary care) of future skin checks was based on the 10‐year risk estimate (see Supplementary Materials). The cut points and tailored recommendations at each risk threshold were based on consensus of dermatologists at the clinic and informed by guidelines^13^ and data on timing of subsequent melanomas.^6^ The preliminary surveillance intervals were then reviewed by the research team against a retrospective audit of 100 patients before being used in this study; the audit found that the intervals correlated well with the actual intervals recommended prior to implementation, except for patients with early‐onset keratinocyte carcinoma, for which clinicians tended to underestimate melanoma risk compared with the risk tool.

The personal risk information presented patients' risk alongside risk‐tailored surveillance recommendations and prevention advice (see Supplementary Materials), which was piloted with three consumers. Patients were also provided with a ‘Melanoma Prevention and Early Detection’ booklet. The study team developed a summary document on the key steps in the project that was printed and displayed in all consultation rooms for clinic staff.

#### 2.2.2 Phase 2: implementation

The risk assessment survey was implemented routinely into clinical practice from February to May 2021. All patients in the waiting room were asked to complete the risk assessment survey using an iPad, regardless of their personal history of melanoma or keratinocyte cancers. Provision of personalised risk and behavioural counselling was conducted predominately by the clinically trained project manager and occasionally by the dermatologists. In a study‐dedicated consultation room, the project manager generated and printed the patient's risk information using an automated process, explained the risk information and provided behavioural counselling on prevention and early detection. The risk information was uploaded to the patient's electronic medical records. Afterwards, the patient attended their usual consultation and reviewed their risk information and risk‐tailored surveillance schedule with their dermatologist.

### 2.3 Data collection

Feasibility was measured as the length of time it took for patients to complete the risk assessment survey, the length of discussion time between the project manager and patient, the integration of the risk information into consultations and any additional burdens for patients and clinic staff arising from the study processes. Feasibility was predominately measured through clinic observations conducted by ALS who recorded field notes.

For measuring acceptability, we were guided by the Theoretical Framework of Acceptability (TFA), which consists of seven constructs: affective attitude, burden, perceived effectiveness, ethicality, intervention coherence, opportunity costs and self‐efficacy.^10^, ^14^ The semi‐structured interview guides explored these constructs and were iteratively revised through regular discussion with the study team to ensure that all relevant themes were explored (Supplementary Materials).

As part of a separate follow‐up survey, patients were asked if they were willing to be contacted to take part an interview about their experience in the clinic receiving their personal melanoma risk information and risk‐based recommendations. Of those who agreed to be contacted, we selectively sampled patients for interview to ensure that we included a balance of gender, age and melanoma risk and they were sent an invitation pack for the interview study (*N* = 37). All clinic staff (doctors, nurses and administrative staff; *N* = 16) were invited to take part in a semi‐structured interview. We conducted the semi‐structured interviews (via Zoom or telephone) until thematic saturation was reached, that is, no new themes were being identified in the discussion.^15^, ^16^ All interviews were audio‐recorded and professionally transcribed.

### 2.4 Data analysis

Qualitative methods have been recommended for assessing implementation‐related outcomes such as feasibility and acceptability from the perspectives of participants, particularly given the capacity of qualitative measures to provide depth to the interpretation of such evaluations.^11^, ^14^, ^17^ We undertook qualitative thematic analysis of the interview transcripts and observational field notes using a coding framework based on the TFA,^10^ supported by NVivo QSR Software. Following methods for reflexive thematic analysis,^18^, ^19^ ALS and BIL coded all transcript data according to the framework categories.^15^ Themes and patterns in the transcript data were then developed and identified within and across the broader categories through consensus between ALS and BIL^18^, ^19^ with any discrepancies resolved through consensus with the study team.^15^ Through data triangulation,^20^ we synthesised the themes from observations, patient and clinic staff interviews and present them here together. We followed the Consolidated Criteria for Reporting Qualitative Studies (COREQ).^21^, ^22^

## 3 RESULTS

A total of 29 patients and 12 clinic staff were interviewed (Table 1). Clinic staff who were not interviewed (*N* = 4) declined due to being away or not being able to find a suitable time.

**TABLE 1:** Participant characteristics.

Patient characteristics	*N* = 29
Age, mean (standard deviation)	66 (11.6)
Female, *N* (%)	16 (55)
Highest level of education, *N* (%)	
High school or equivalent	4 (14)
Trade/diploma	10 (35)
University degree or higher	14 (48)
Ten‐year risk category, *N* (%)	
75%–100%	6 (21)
50%–74%	3 (10)
25%–49%	2 (7)
0%–24%	18 (62)
Personal history of melanoma, *N* (%)	
Yes	18 (62)
Staff characteristics	(*N* = 12)
Dermatologist	6
Allied health staff (melanographer)	2
Clinic administrative staff	3
Software developer	1

The risk assessment process and provision of risk‐tailored surveillance schedules in this skin cancer focussed dermatology clinic was feasible. Adjustments were necessary to accommodate the study processes in the clinic, such as a dedicated room for the behavioural counselling. Although staff did not feel that the workflow was disrupted, some did suggest that the process may be improved if patients completed the survey online prior to attending the clinic. The risk assessment survey was provided routinely, and patients completed it in a timely manner, on average, taking 11 min (range: 5–27 min); it should be noted that more than half the questions in the survey were related to clinical and research questions rather than required for the generation of the personal risk estimate. The risk communication and behavioural counselling discussions between the project manager and patients lasted on average 5 min (range: 3–14 min). No follow‐up to the risk assessment process after their appointment was required; however, patients were asked at their next visit at the clinic whether they had any changes to their risk factors (e.g., skin cancer diagnosis) and if so, they were asked to complete the risk assessment survey again.

Information technology‐related barriers related to the risk assessment that arose in real time were mitigated by the onsite informational technology and software support team. Findings from the semi‐structured interviews were categorised according to the TFA (see Supplementary Materials) and are discussed in more detail below.

From the perspective of clinic staff, having a dedicated project manager to run the day‐to‐day project was highly valued and identified as a key facilitator for successful implementation:

I think you need a dedicated person that doesn't have other roles in the clinic. That person needs training. But in our case she didn't need much training, because she was already excellent. But if you were rolling it out in a different centre you would need more specific training (ID7, clinic staff)

**FIGURE 2:**
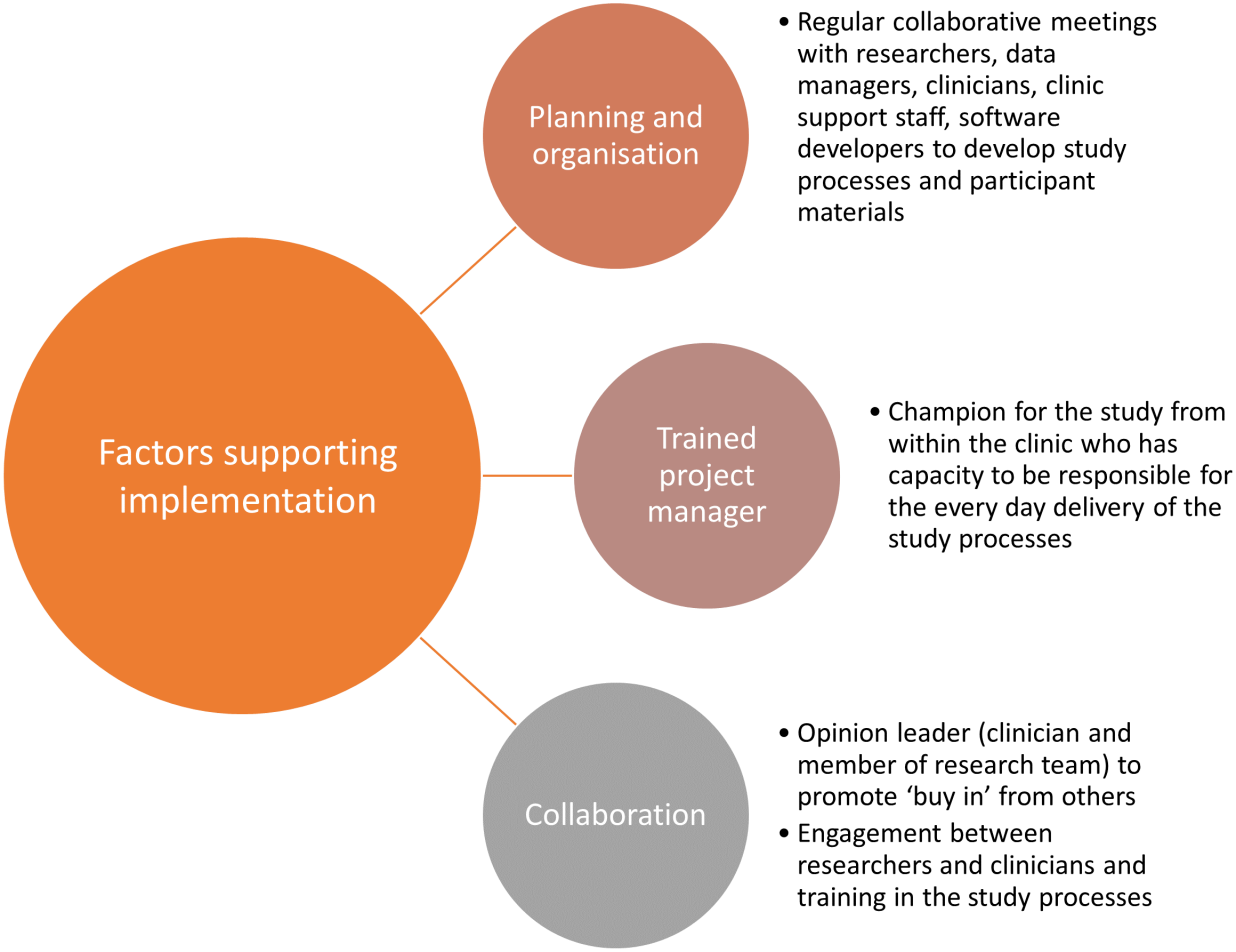
Key factors supporting the study implementation from the perspective of clinic staff.

Other facilitators supporting the feasibility of the study included regular collaborative meetings, comprehensive planning and organisation in the pre‐implementation phase, and clinician researchers who acted as champions for the delivery of the study (Figure 2). For both patients and clinic staff, having the iPad‐based survey completed in the waiting room was viewed as a practical use of time, particularly because the purpose of the risk assessment process was perceived to align with improved care and the research culture at the Melanoma Institute Australia:

[Patients] know that it's a research institution so they're expecting us to give them news and updated technologies and information. So when we say, oh, we developed a way to calculate your risk, I feel that they [feel] okay, so I'm in the right place (ID02, clinic staff)

Patients were generally interested in their risk information and valued receiving the feedback in‐person immediately after completing the survey. Another facilitator was that completing the survey via iPad was straightforward and patients felt that their risk information was easy to understand. For most patients, they felt that the risk information was affirming and validated their need for follow‐up surveillance and sun protection strategies:

I thought it was terrific, I did actually. Reminded me to wear my hat and sunglasses, which I know is on every bit of literature that comes out. But anyway, it's a good reminder. (ID48, patient)

The behavioural counselling was described as confirming their current knowledge and behaviours, yet it was still highly valued and some patients learned new information:

So that was pretty useful because I think one of the questions was how often do you put sunscreen on? I was like well, I'm really good when the sun's shining type thing. It was like no, you need to be really good. Even if it's cloudy in Australia. So, I was educated on that. (ID43, patient)

Many patients who received a low risk estimate described a sense of relief:

I just remember feeling a sense of relief when she told me. She said, what do you think your risk level is? I plucked out a number. What she came back with was heaps lower. I thought that's great. Awesome. I just thought, wow. (ID12, patient)

But when patients' perceived risk was discordant with their risk estimate, particularly if the risk estimate was lower than expected, instead of relief, some patients reported experiencing confusion, worry and mistrust in the risk information:

I expected to be higher… I probably should have asked how did you come at the figure of six per cent with the little bit that I gave you? I don't need to know but I thought that was a question I should have asked. Because I'm now being expected to believe that figure but I don't know where you got that figure. Now I will believe it because I trust you (ID135, patient)

Among the patient group with discordant risks, particularly for those whose risk estimate was lower than what they expected, some reported a reluctance to reduce their clinical skin check frequency and to be discharged from the dermatology clinic to the care of a general practitioner in the primary care setting:

Doctor did say to me last time, when I had that two per cent [risk score], she said, you don't need to come back anymore… But I said to her well do you mind if I still come back and she said no, that's okay you can still come back. Because I had told her that I do suffer from things—anxiety (ID152, patient)

Another facilitator for the implementation of this intervention was that patients' sense of trust and confidence in the care provided by the dermatology clinic was high, but many reported having distrust in mainstream general practice and skin cancer primary care clinics for skin cancer surveillance, which was also observed by clinic staff. This distrust highlights a potential barrier as it fed into the reluctance to reduce surveillance frequency or be discharged from the dermatology clinic for those at low risk:

But a lot of it is probably a bit of anxiety. But also (…) if they are super low risk and you say oh you can go back to your GP. You don't have to see us regularly a lot of them are like, we don't have a GP. I don't have a regular GP and even if I go see a GP how am I going to find a good GP? I'm already here seeing you. (ID5, clinic staff)

Clinic staff reported positive attitudes towards the integration of the risk assessment process and risk‐based surveillance schedules into their routine practice. The risk assessment provided an objective evidence‐based means to determine patients' risk, which helped to justify surveillance recommendations and enhanced their confidence in providing these:

It is easier for me to explain to the patient how high the risk is, because I have a number. Also it's a good reason for them to understand why I'm saying that he or she needs to come back in four months or six months. It's just like supporting my clinical decisions with the patient. (ID9, clinic staff)

Generally, clinicians reported having trust in the risk estimate but identified some lack of clarity for their patients regarding the potential for the risk estimate to change in future, and confusion for those whose risk information didn't align with their expectations. Some clinicians felt that the risk information might help mitigate patient anxiety, but also reported that patients experienced feelings of guilt about past sunburn and sun habits, prompted by the risk information. Some clinicians reported situations when the risk estimate did not appear accurate; for example the risk tools would underestimate risk for patients with severe dysplastic nevus syndrome or with several relatives with multiple primary melanomas because the risk tools only asked if a person had ‘none, few, some or many moles’ and whether they had a first‐degree relative with melanoma.

## 4 DISCUSSION

This study showed high feasibility of integrating personal melanoma risk assessment using an iPad and risk‐tailored skin surveillance alongside preventive behavioural counselling into routine practice at a large, busy skin cancer focussed dermatology clinic. The stepwise implementation strategy supported continuing collaboration between researchers, clinicians, clinic and technical support staff, which allowed for real‐time resolution of day‐to‐day issues and iterative improvement of risk provision processes and patient materials as needed. Some staff suggested completing the risk assessment prior to attending the clinic. Overall, the risk assessment and communication of melanoma risk information including risk‐based screening and surveillance recommendations were perceived to form part of improved patient care. Further considerations for the implementation of systematic melanoma risk assessment and risk‐tailored surveillance schedules are discussed below.

This study is the first, to our knowledge, to explore the acceptability of providing risk‐tailored skin surveillance schedules based on a validated risk assessment as part of routine care in dermatology clinics. For patients, key facilitators included receiving their personal risk information immediately post‐survey completion and in‐person, which they found highly acceptable. Patients reported that completing the survey while in the waiting room was a good use of their time and was convenient. Other studies have found similar facilitators to participating in risk assessment in risk‐based screening programs, such as ease of access.^23^ Financial burden, mode of risk assessment (including location of where the assessment takes place) and perceived effort are reported factors that contribute to perceived accessibility,^24^ which were addressed in our study by incorporating the risk assessment survey into routine care.

For clinic staff in our study, facilitators for implementing the study processes included comprehensive planning and organisation, regular collaboration throughout the study and a trained clinician–researcher project manager running the study in the clinic. Future research studies could examine more streamlined risk assessment processes such as the patient completing the risk assessment prior to attending the clinic and the personalised risk information could be discussed with the dermatologist in their usual consultation. Other studies that have implemented risk stratification within cancer screening programs have found that health professionals are concerned about risk‐based approaches raising more patient queries and necessitating more time for consultations, which might exacerbate existing time constraints and heavy workloads.^25^ However, in our study we found that the risk assessment survey was easy to complete, and few barriers were identified to implementing it in real‐time, routine clinical practice at no additional cost to the patient.

Providing risk information that is contradictory to existing risk perception is a challenge for risk communication. One theme identified in our study was that receiving a risk estimate that was lower than expected could promote initial confusion or worry. This finding aligns with other research evidence that receiving a lower estimate than expected may prompt concern,^26^ particularly if the corresponding recommendation is to decrease screening, which a patient might have already been engaged in for many years.^27^, ^28^ Previous studies exploring the hypothetical provision of risk‐tailored cancer screening including for melanoma have shown lower acceptability for reducing or stopping screening for low‐risk groups.^23^, ^29^, ^30^ Engaging in skin cancer screening may be perceived as part of taking responsibility for one's own health and people may be reluctant to give up screening if they are already having regular skin checks.^30^

Our findings also highlight a need to further explore how to support informed choice for patients regarding reducing the frequency of skin checks where appropriate to improve acceptability for lower risk patient groups.^27^ There is growing research on the acceptability of risk stratified approaches, including increasing intervals of screening.^23^, ^25^ Educational strategies such as providing easy to understand information on the safety of reduced screening frequencies, including details regarding the accuracy of risk estimates and the potential for these to change over time, have been recommended.^27^, ^31^, ^32^, ^33^ Given that patients in our study highly valued the face‐to‐face conversions with clinicians about their risk, this may be an opportunity to build patient confidence in low‐risk results by providing additional education regarding the potential benefits of reducing skin surveillance, offering behavioural counselling on skin self‐examination and primary prevention behaviours (as done in this study), or offering teledermatology for lesions of concern. Acceptability may be further improved by ongoing improvements in risk assessment tools that more comprehensively capture skin cancer risk factors, and by ensuring that behavioural counselling addresses patient guilt about past sunburns and sun habits that were prompted by completing the risk assessment survey. Future research could also explore whether intervention feasibility and acceptability varies according to personal melanoma risk and whether or not patients are already engaged in regular skin checks.

There is consensus that more targeted approaches to melanoma screening and surveillance that are directed to those most at risk may better balance the benefits and potential harms of screening for melanoma.^3^, ^9^ The barriers and facilitators to implementation identified in our study represent an important step in understanding how risk‐based approaches to cancer screening could be incorporated into clinical care. The strengths of this study lie in the prospective implementation theory‐based study design, which enabled an in‐depth evaluation of the feasibility and acceptability. The use of validated risk prediction tools and evidence‐based risk communication and behavioural counselling are other strengths.

Limitations to the generalisability of our findings include the patient cohort, which may be more motivated and engaged with screening than the broader population. Patients that attend the Melanoma Institute Australia may also be more used to providing data for research studies, and they expressed high levels of trust in the clinicians. These factors likely supported the successful implementation of the study and improved patient acceptability. Another limitation was that we were not able to measure specifically how many patients did not complete the risk assessment survey in the waiting room, although feedback from the clinic staff indicated that the vast majority of patients completed it. Finally, the risk‐based thresholds and corresponding follow‐up intervals that were used in this study were based on limited evidence and require further evaluation.

## 5 CONCLUSIONS

In conclusion, this study showed high feasibility of integrating personal melanoma risk assessment using an iPad and the provision of risk‐tailored surveillance schedules alongside preventive behavioural counselling into routine practice at a large skin cancer focussed dermatology clinic. Overall, the processes for risk assessment and provision of tailored surveillance recommendations were perceived to enhance patient care.

## AUTHOR CONTRIBUTIONS

**A. L. Smith:** Conceptualization (equal); data curation (lead); formal analysis (lead); investigation (lead); methodology (lead); supervision (equal); writing – original draft (lead); writing – review and editing (lead). **A. K. Smit:** Data curation (equal); formal analysis (equal); investigation (equal); methodology (equal); project administration (equal); supervision (equal); visualization (equal); writing – original draft (lead); writing – review and editing (lead). **B. I. Laginha:** Data curation (supporting); formal analysis (supporting); writing – original draft (supporting); writing – review and editing (supporting). **N. Singh:** Formal analysis (supporting); writing – original draft (supporting); writing – review and editing (supporting). **B. Gallo:** Conceptualization (supporting); data curation (supporting); investigation (equal); project administration (equal); supervision (equal); writing – original draft (supporting); writing – review and editing (supporting). **L. Martin:** Conceptualization (lead); data curation (lead); formal analysis (equal); funding acquisition (lead); investigation (equal); methodology (equal); project administration (equal); resources (equal); supervision (equal); writing – original draft (equal); writing – review and editing (equal). **A. E. Cust:** Conceptualization (lead); data curation (lead); formal analysis (equal); funding acquisition (lead); investigation (lead); methodology (lead); project administration (lead); resources (lead); supervision (lead); visualization (equal); writing – original draft (equal); writing – review and editing (equal).

## FUNDING INFORMATION

This project was funded by grants from the NHMRC (Centre of Research Excellence #1135285 and Investigator Grant to AEC #2008454) and Sydney Catalyst Translational Cancer Research Centre. AKS is supported by a NHMRC Synergy grant (#2009923). LKM is funded by the Warwick L Morison Professorship of Dermatology, UNSW. The funders had no role in study design, data collection and analysis, decision to publish, or preparation of the manuscript.

## CONFLICT OF INTEREST STATEMENT

The authors have no conflicts to declare.

## ETHICS STATEMENT

Sydney Local Health District Ethics Review Committee (X18‐0426, HREC/18/RPAH/606).

## PATIENT CONSENT

All participants provided written informed consent.

## Supporting information


Tables S1–S4.

## Data Availability

The data from this study are available on request, subject to ethics committee and governance approvals.
