# Supplementary material for: Implementing systematic melanoma risk assessment and risk‐tailored surveillance in a skin cancer focussed dermatology clinic: A qualitative study of feasibility and acceptability to patients and clinic staff
Source: Cancer Med. 2024 Feb 4;13(2):e6976. doi: 10.1002/cam4.6976 (PMC10839129; doi:10.1002/cam4.6976)
Supplement: Supplementary file 1 — Tables S1–S4. [file CAM4-13-e6976-s001.docx]

**Supplementary Materials**

Supplementary Table 1. Semi-structured interview guide for **clinic staff interviews** (feasibility and acceptability questions according to Theoretical Framework of Acceptability constructs)

| **Construct^1^** | **Flow of discussion and key questions** | Additional prompts |
| --- | --- | --- |
| Affective attitude | Can you tell me how you feel about using the iPad-based risk assessment / delivering the risk assessment, tailored education and surveillance in the clinic? | - Was there anything in particular that you liked / disliked about the process? - Is there anything you would change? |
| Ethicality | Does providing this personalised information to patients help you to do something that is important to you? In what way? | - Do you believe providing personal risk assessment and tailored education and skin surveillance is the right thing to do? |
| Burden | How much of a burden is it for you to use / provide personal risk assessment and tailored education and skin surveillance in the clinic? | - In your opinion, how easy or difficult was it to implement this in the clinic? - [Clinicians only: How easy or difficult was it to comprehend the personal risk report?] - [Clinicians only: Can you tell me how long it took to explain the risk estimate, provide tailored education, discuss the surveillance schedule with the patient?] |
| Self-efficacy | How confident are you in providing personal risk assessment and tailored education and skin surveillance in the clinic?  Can you tell me if anything has impacted on your confidence in delivering it? | - E.g. the materials (the iPad; the printed personal risk report / tailored education / tailored surveillance schedule)? - E.g. the time you have available? |
| Opportunity cost | How does the delivery of this impact on your workload?  Why would you continue to provide it? To not provide it? | - On the number of patients seen? - Is there anything that it stops you from doing that you would otherwise do? |
| Intervention coherence | Do you have a sense for why this information is being provided to patents in the clinic?  Do you think the information is helpful for them? | - What do you think is the most important aspect of the information (risk factor collection, risk estimate, prevention advice, skin check advice)? |
| Global acceptability | Overall, to what extent do you find the process of providing patients’ personal risk information and tailored advice acceptable? | - Did you experience any problems, e.g. scheduling follow-up appointments, assisting patients in finding an alternative doctor (e.g. if patient has been advised they do not need to come back to dermatology clinic for skin surveillance)? |

^1^ Constructs are based on the Theoretical Framework of Acceptability; Sekhon et al. 2017

Supplementary Table 2. Semi-structured interview guide for **patient interviews** (feasibility and acceptability questions according to Theoretical Framework of Acceptability constructs)

| **Construct**^1^ | **Flow of discussion and key questions** | **Additional prompts** |
| --- | --- | --- |
| Affective attitude | Can you tell me how you feel about completing the risk assessment questions on the iPad? | - Was there anything in particular that you liked / disliked about the process of completing the questions? - Is there anything you would change? |
| Burden | How much of a burden was it for you to complete the questions on the iPad in the clinic?  In your opinion, how easy or difficult was it to complete the risk assessment questions? |  |
| Self-efficacy | How confident were you in answering the risk assessment questions on the iPad?  Can you tell me if anything impacted on your ability to complete the questions? | - E.g. the time you had available in the waiting room? |
| Affective attitude | Can you tell me how you felt about receiving your personal melanoma risk estimate and report?  Can you tell me how you felt about receiving your sun protection and skin check advice?  What advice did you receive about when and where to have your next skin check?  How did you feel about receiving this advice? (e.g. Concerned? Reassured?) | - How did you find talking with the [doctor/nurse/health professional] about your melanoma risk and future skin checks? |
| Opportunity cost | Is there anything that you have been advised to do that would be a change from your usual skin check or sun protection routine? | - E.g. No longer seeing specialist for check-up (if they are told they are low risk and should see GP for future check-ups) |
| Intervention coherence | Do you have a sense for why the personal risk report is being used in the clinic?  Do you think the information is helpful for you? | - In your opinion, to what extent do you think your personal risk report will make a difference to your behaviour (e.g. checking your own skin; sun-protective behaviours; how often you have a skin check)? |
| Global acceptability | Overall, to what extent did you find the process of receiving your personal risk information and tailored advice acceptable? | Was the process satisfactory? |

^1^ Constructs are based on the Theoretical Framework of Acceptability; Sekhon et al. 2017

**Personal risk of developing a subsequent melanoma**

**
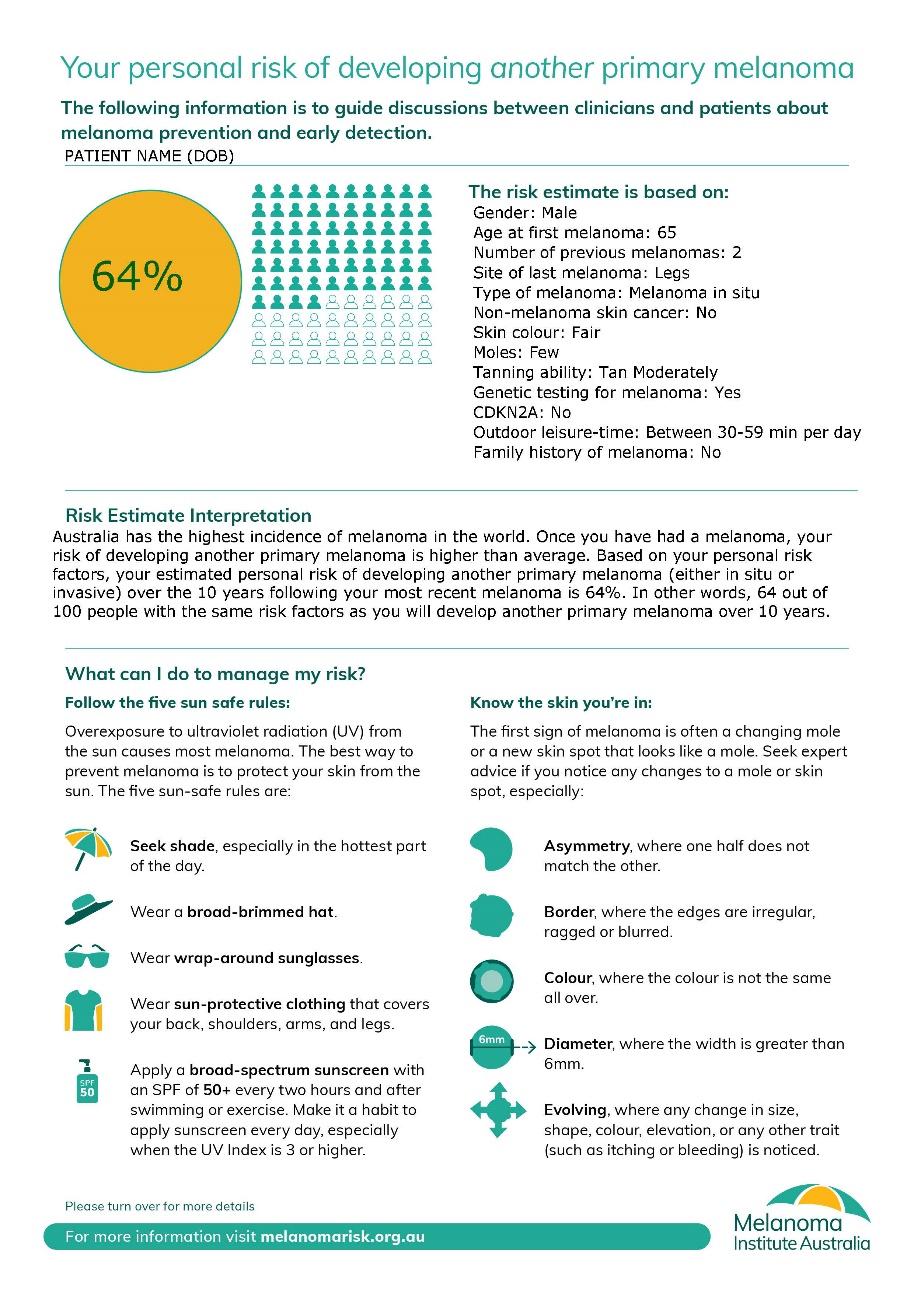

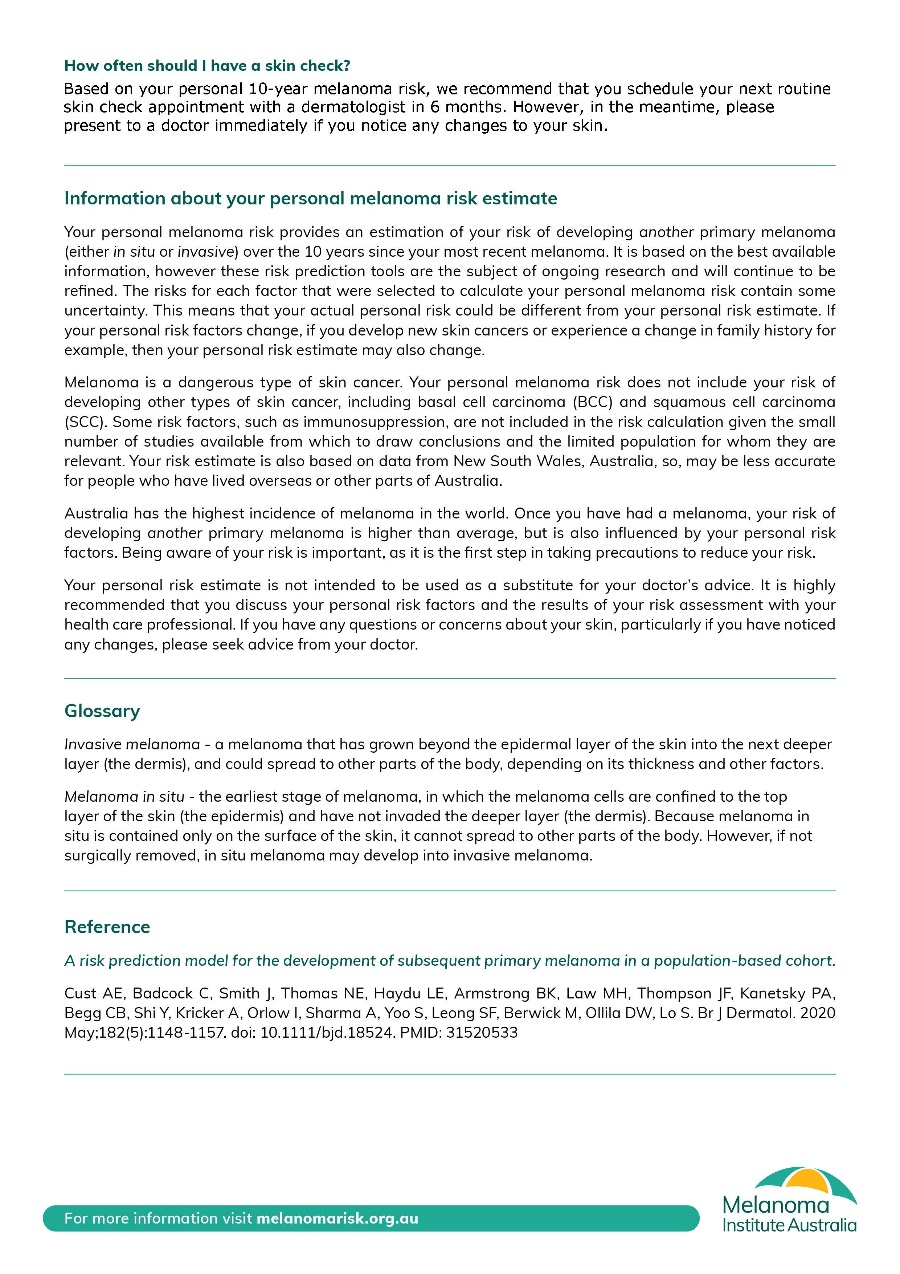
**

**Personal risk of developing a first primary melanoma**

**
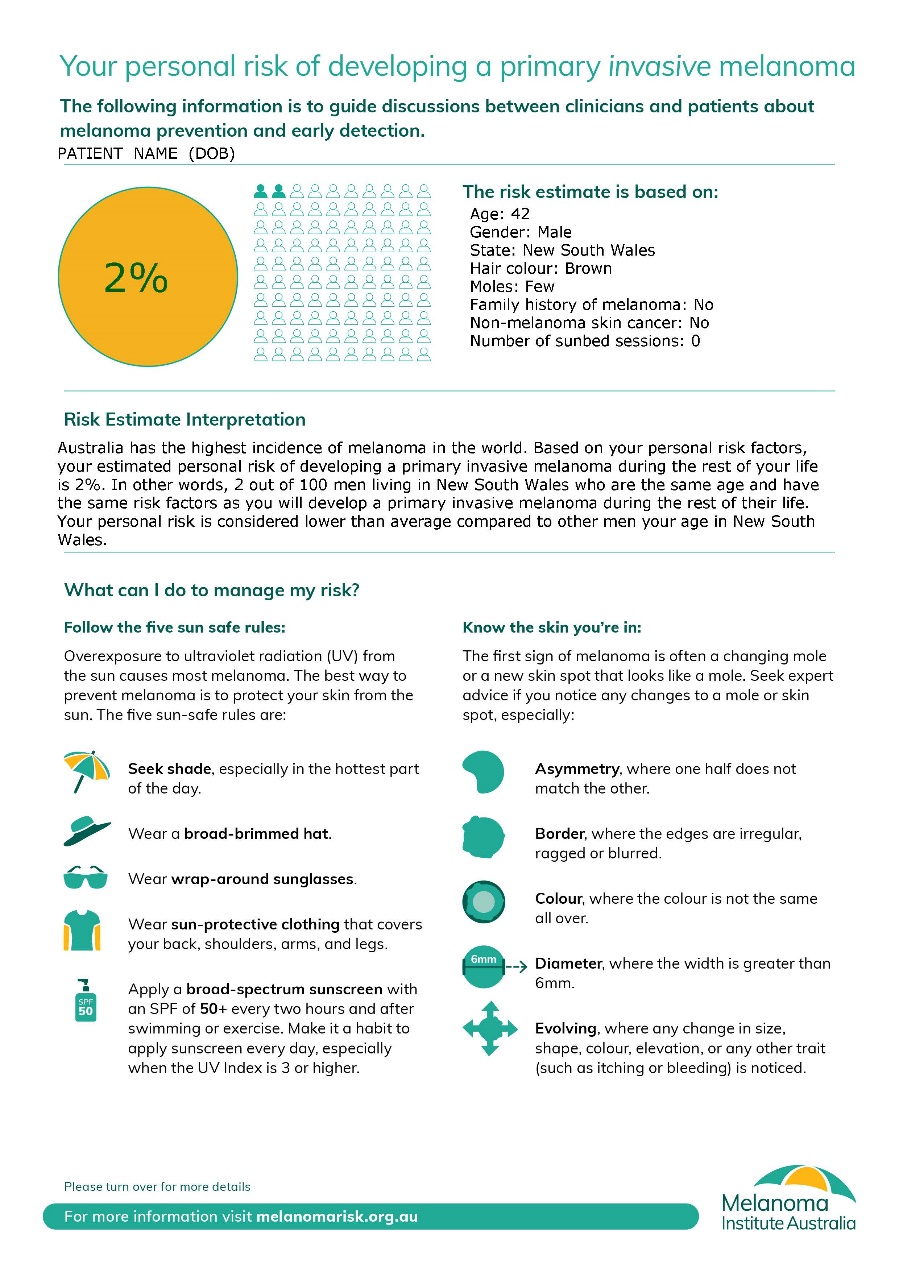

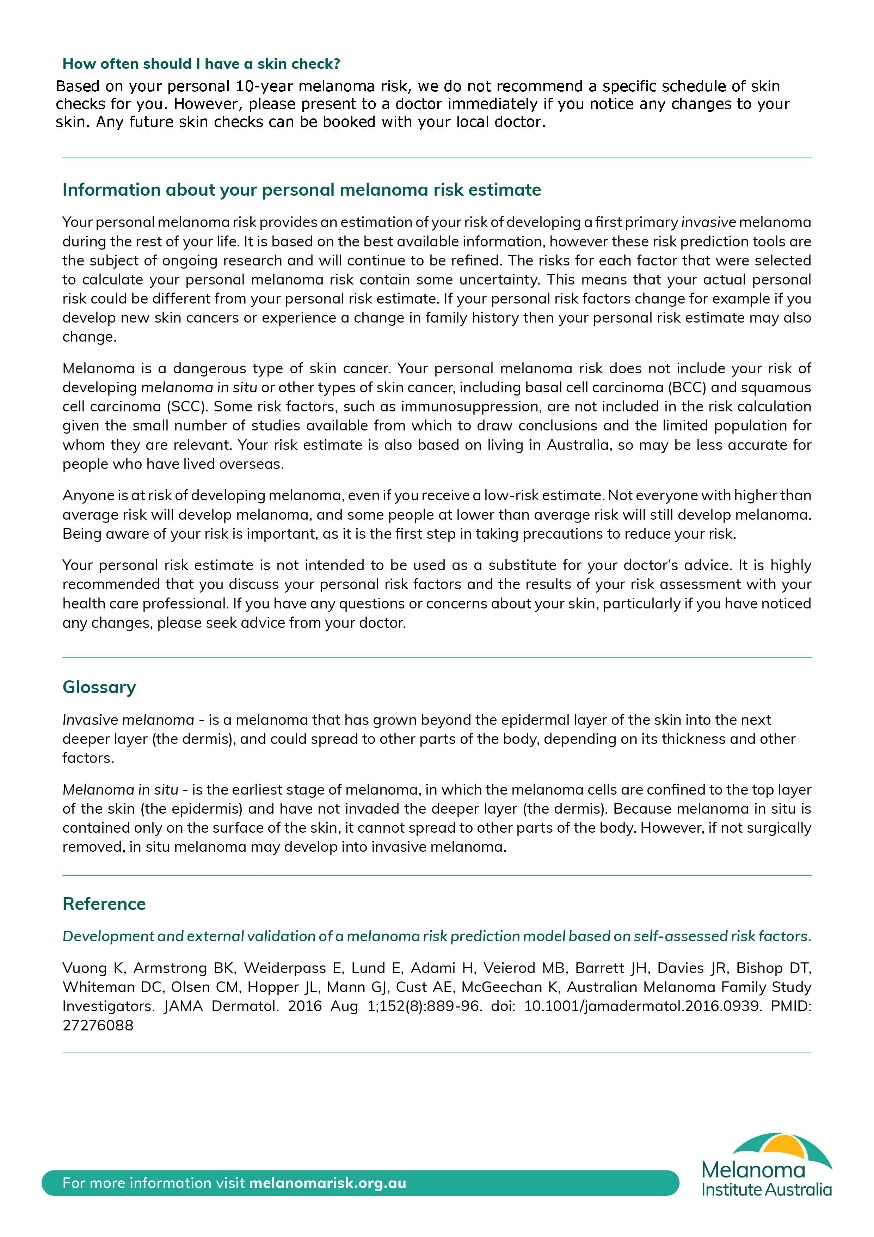
**

**Supplementary Table 3.** Risk thresholds used to determine screening and surveillance schedules

| Ten-year risk threshold | Corresponding statement on personalised risk printout | Patient directed to: |
| --- | --- | --- |
| ≥75.0% | Based on your personal ten-year melanoma risk, we recommend that you schedule your next routine skin check appointment with a dermatologist in 4 months. However, in the meantime, please present to a doctor immediately if you notice any changes to your skin. | MIA dermatology clinic |
| 25.0-74.9% | Based on your personal ten-year melanoma risk, we recommend that you schedule your next routine skin check appointment with a dermatologist in 6 months. However, in the meantime, please present to a doctor immediately if you notice any changes to your skin. | MIA dermatology clinic |
| 10.0-24.9%, OR <10.0% and multiple severely atypical naevi* | Based on your personal ten-year melanoma risk, we recommend that you schedule your next routine skin check appointment with a dermatologist in 1 year. However, in the meantime, please present to a doctor immediately if you notice any changes to your skin in the meantime. | MIA dermatology clinic |
| <10.0% and has a previous melanoma | Based on your personal ten-year melanoma risk, we recommend that you schedule your next routine skin check appointment with your local doctor in 1 year. However, in the meantime, please present to a doctor immediately if you notice any changes to your skin. | Discharge to GP or skin cancer clinic or community dermatologist |
| <10.0% and no previous melanoma | Based on your personal ten-year melanoma risk, we do not recommend a specific schedule of skin checks for you. However, please present to a doctor immediately if you notice any changes to your skin. Any future skin checks can be booked with your local doctor. | Discharge to GP or skin cancer clinic or community dermatologist |

MIA, Melanoma Institute Australia; GP, general practitioner

*dysplastic naevus syndrome (many atypical moles)

The risk thresholds and associated recommendations were devised by the study team and are subject to change.

**Supplementary Table 4.** Key themes from the semi-structured interviews according to Theoretical Framework of Acceptability constructs

| **Theoretical Framework of Acceptability Construct** | **Definition of the constructs* for the Tailored Surveillance Project** | **Illustrative quotes from the semi-structured interviews in the Tailored Surveillance Project** |
| --- | --- | --- |
| Affective attitude | How clinic staff and patients felt about the risk tailored processes in the Tailored Surveillance Project | *“[the risk assessment] was pretty simple. Self-explanatory. The lady who gave me the iPad said if I had any questions or I wasn’t sure, just to ask. So yeah, it was fine.”* (Patient) |
| Burden | The perceived amount of effort that is required for the risk tailored processes | *“To be honest, when you’re sitting in a waiting room waiting, there’s not a lot of other things to keep you occupied so it was non-intrusive.”* (Patient) |
| Ethicality | How well the risk tailored processes align with individuals’ values | *“[Patients] know that it’s a research institution so they're expecting us to give them news and updated technologies and information. So when we say, oh, we developed a way to calculate your risk, I feel that they [feel] okay, so I’m in the right place”* (Clinic staff) |
| Intervention coherence | The extent to which patients and clinic staff understand the risk tailored processes and how they work | *“I just did think that the percentage was a bit low for what I think for myself - for my own body (…) It was good to have it (the risk report) and run through it (…) I read all the way through it. But in the end, I suppose, once I had read everything and I read the pamphlets that I was given, the risk assessment though, I did then just throw it away because I thought, okay, I don’t quite agree with that.”* (Patient) |
| Opportunity costs | Benefits, resources or principles that must be given up to participate | *“We could probably give it to more patients and ask more patients to do the risk assessment, but, as I said, it needs - we need someone who's going to sit down with a patient, explain what the whole thing is about in reception admin; we don't have time for that”* (Clinic staff) |
| Perceived effectiveness | How likely patients and clinic staff feel the risk tailored approaches is to achieve its goal | *“I think there's been a couple of - at least a couple of patients where I've been like, oh I'm with a doctor and I'm like I'm not sure how often to see them. They were like have they done the risk prediction? No they haven't. Well let's just quickly calculate it anyway outside the study to guide how often we should see them. There are definitely patients that are - yeah where we're not sure how often we should see them. Yeah that has helped.”* (Clinic staff) |
| Self-efficacy | How confident patients and clinic staff are about participating in the risk tailored processes | *“I think it is straightforward. I just feel that the numbers talk more than the words but that’s a number thing, right.”* (Clinic staff) |

*Definitions of the component constructs based on the Theoretical Framework of Acceptability (TFA; Sekhon et al., 2017)
